# NEO E-vita—NEO era!

**DOI:** 10.1007/s12055-021-01303-0

**Published:** 2022-01-19

**Authors:** Heinz Jakob, Fanar Mourad

**Affiliations:** 1 Department of Cardiothoracic Surgery, West German Heart and Vascular Center, University Hospital Essen, Essen, Germany; 2 Diagnosticum Mülheim, William-Shakespeare-Ring 9–11, 45470 Mülheim, Germany

**Keywords:** Frozen elephant trunk, Total arch replacement, E-vita NEO, Thromboelastometry

## Abstract

In this review article, the history of the first commercially available thoracic aortic hybrid graft, the E-vita Open, later modified to the blood-impermeable E-vita Open Plus, is reported from its beginning in 2005 until its newest variation, the E-vita Open NEO, European conformity (CE) marked in 2020. Besides the background of its design and clinical experience in Essen, concomitant evolutionary steps in surgery as well as in strategic approaches like the hybrid operating room concept are displayed, finally leading to a well-rounded surgical package with a device that can be applied in all elective as well as emergency situations with complex arch involving aortic pathologies. With the E-vita Open NEO, now, surgery has been facilitated to convenient anastomosing in any of the arch zones, with the opportunity to use the island technique with a straight graft variation as well as individual head vessel anastomosing with either a trifurcated graft for zone 0 or the branched graft for zone 2 or 3 implantation. With its proven long-term stability, the surgical armamentarium to cope with complex multisegmental thoracic aortic pathologies has been significantly improved.

Based on early experience with many redo cases after proximal repair of type I aortic dissection, the operative Essen program using covered stent grafts (Talent, Medtronic, Meerbusch, Germany) started in June 2001 to treat arch and descending aorta aneurysm formation or distal malperfusion, by inserting the reversed mounted stent graft onto the introducer, through the resected aortic arch into the true lumen (TL) of the descending aorta [1].

Due to a dismal experience with the stiff introducer and the creation of proximal endoleaks, caused by penetrating bare springs, despite stay sutures for fixation within the new arch prosthesis, the program was stopped after 14 cases. Based on our conception, a one-piece hybrid graft, without bare springs, was designed and a clinical prototype was produced by JOTEC (Hechingen, Germany) in 2004 (Fig. 1). In 2005, this hybrid graft became the first commercially available hybrid vascular prosthesis in Europe, later called frozen elephant trunk (FET), thus initiating the era of intended one-stage repair of complex thoracic aortic disease involving the arch [2, 3]. Due to the fact that “mad cow disease” in those days prohibited coating of the new device with bovine collagen or gelatine, as well as the intended invagination of the arch part into the stented portion within the minimized introducer, the woven prosthesis had to be preclotted with fibrin sealant (Immuno AG, Vienna, Austria, later Baxter, Höchstadt, Germany) (Fig. 2). Though efficient, primary blood impermeability became the next step forward and was achieved by a tighter weaving process. This could be demonstrated in animal experiments and first clinical application, leading to the E-vita Open Plus product, which was then unanimously accepted by the surgical community in 2008 [4] (Figs. 3 and 4).

**Fig. 1:**
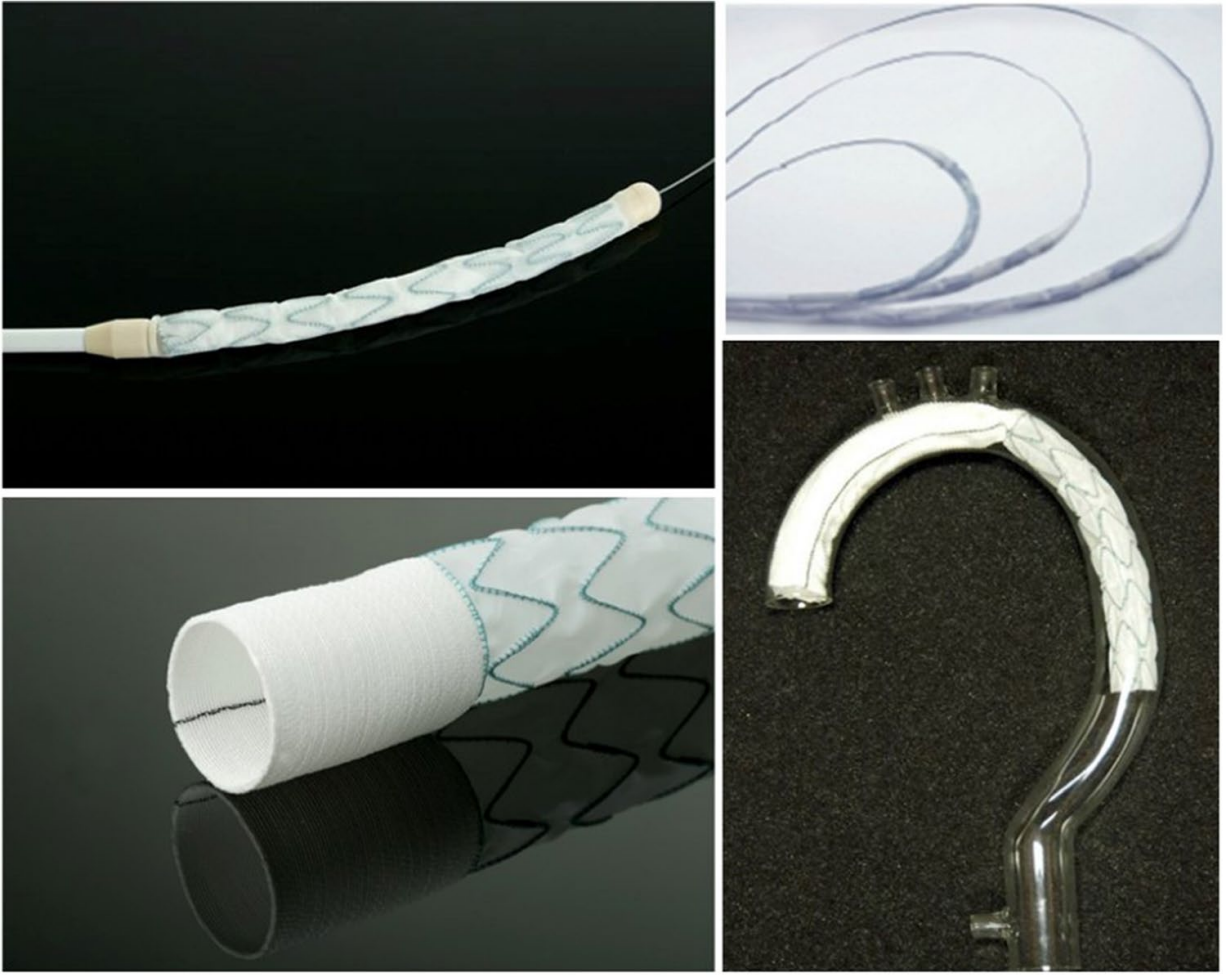
E-vita Open (1^st^ generation)

**Fig. 2:**
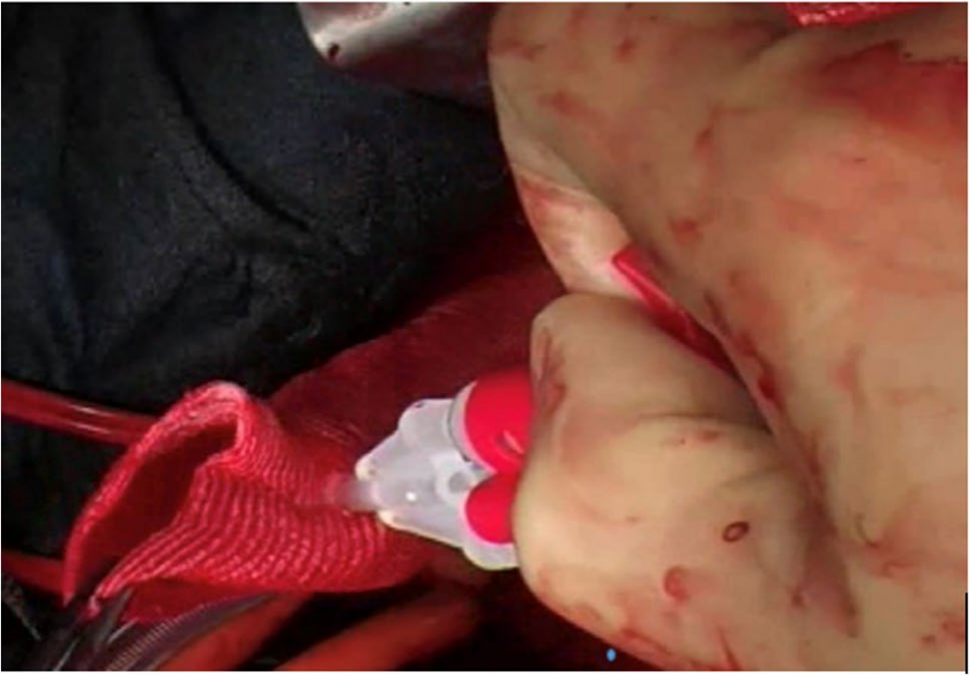
The pre-clotting of E-vita Open with fibrin sealant

**Fig. 3:**
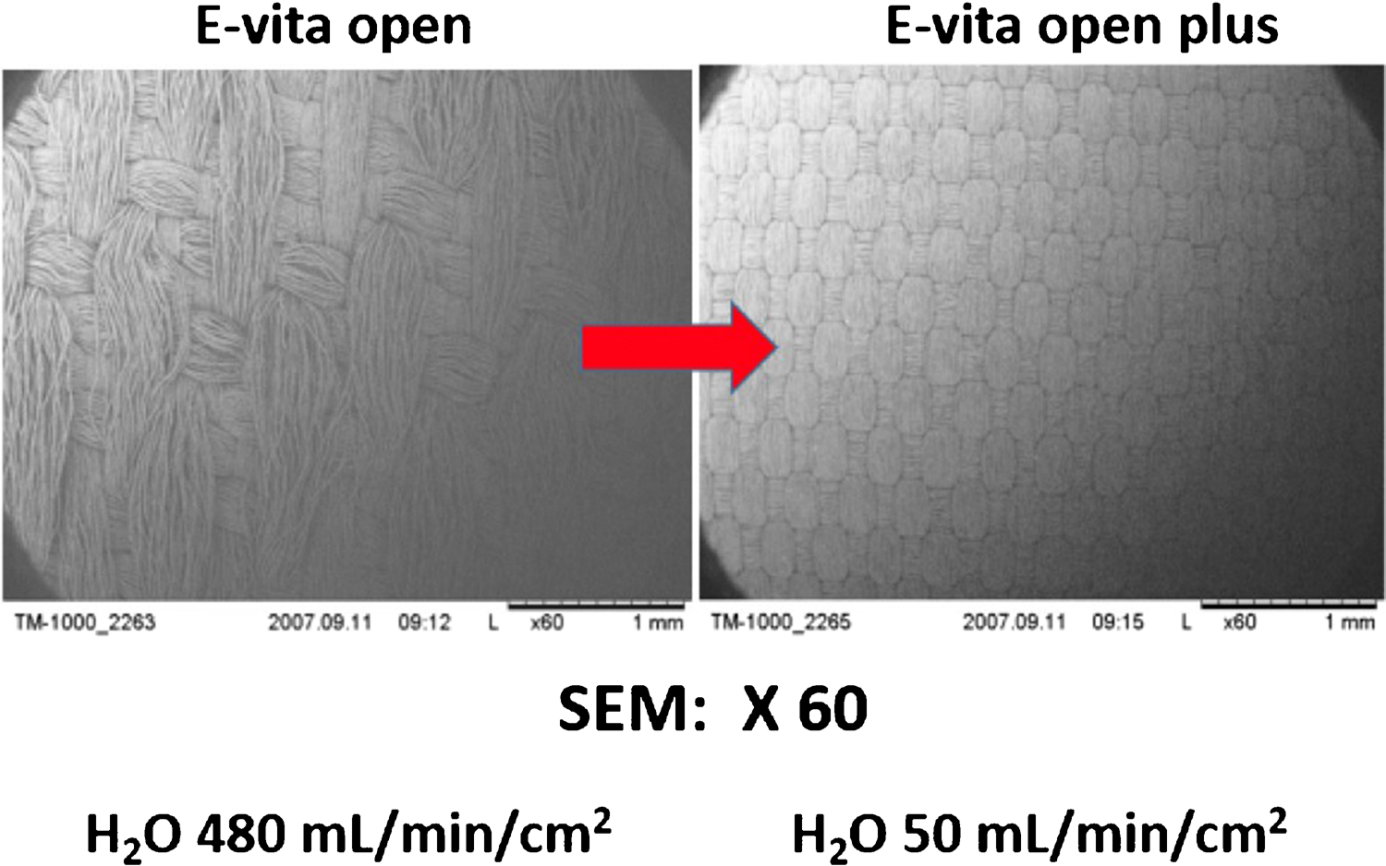
Animal experiments show low water porosity in the E-vita Open Plus in comparison to E-vita Open (1^st^ generation)

**Fig. 4:**
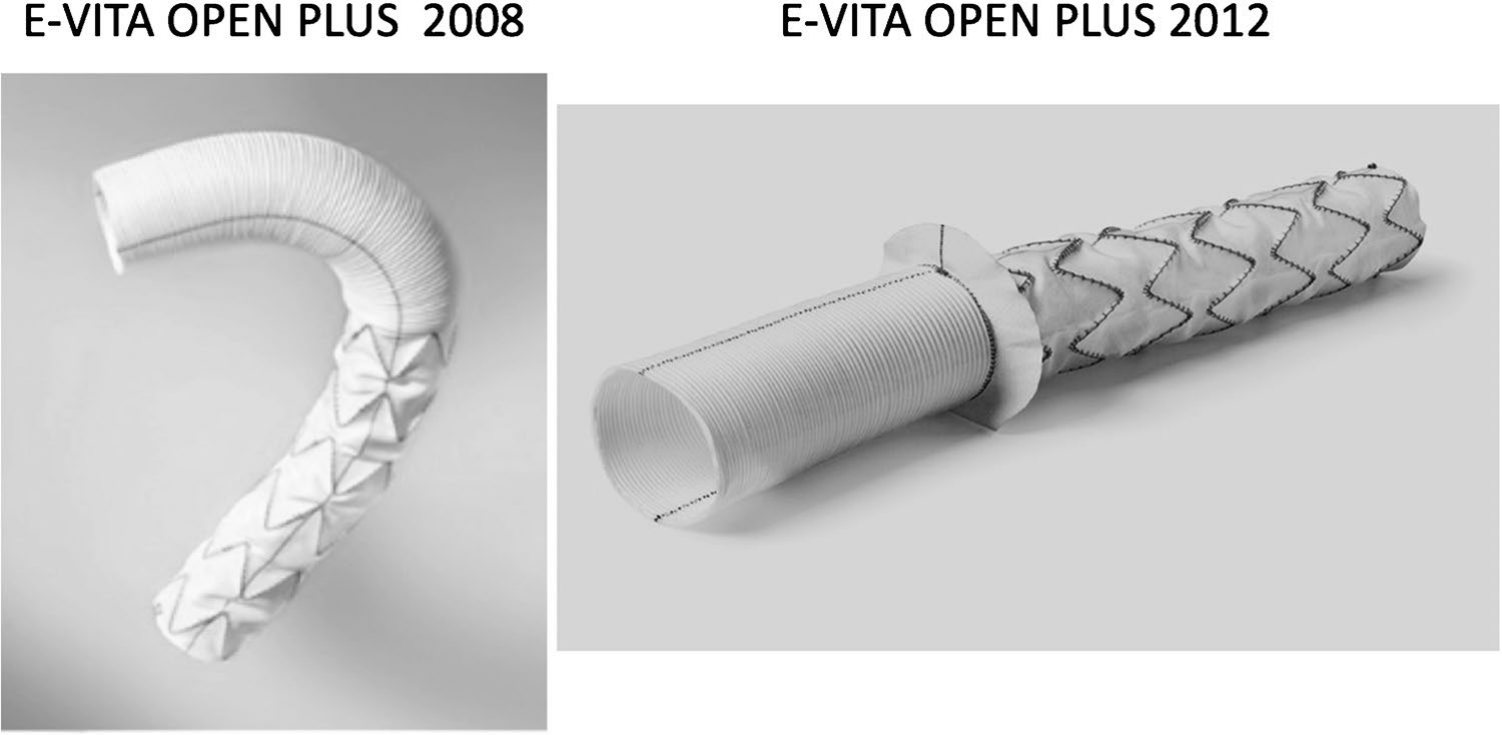
The incorporation of a sewing cuff to E-vita Open Plus

In parallel, several additional developments in surgical strategies took place (Table 1). These include important concomitant innovations like the introduction of our hybrid room concept in 2004, enabling simultaneous diagnostics and therapy on one table [5, 6]; the anesthesiological improvements in coagulation management by rotational thromboelastometry in 2005 [7]; the direct true lumen cannulation in disastrous acute type A aortic dissections in 2006 [8]; and the institution of angioscopy for guidance of the FET into the true lumen of the invisible descending aorta in aortic dissection and control of its positioning [9].

**Table 1:** Surgical strategy and device development in Essen

	1st application	Reference
Arch repair + TEVAR antegrade	2001	EJCTS 2002 [1]
Hybrid room concept	2004	Herz 2011, EJCTS 2013 [5, 6]
E-vita Open implantation Combining classic surgery with descending stent grafting	2005	Herz 2005 [2] Ann Thorac Surg 2008 [3]
Thromboelastometry-directed coagulation management	2005	Transfus Med Hemother 2012 [7]
Direct true lumen asc. aorta cannulation	2006	JTCVS 2007 [8]
Angioscopy—aortic disease and landing zone evaluation	2007	EJCTS 2010 [9]
E-vita Open Plus	2008	J Endovasc Ther 2010 [4]
Stent graft length reduction to 130 mm Proximalization of distal anastomosis to zone 2	2008/2009	EJCTS 2012 [14, 16]
International E-vita Open registry	2008	J Cardiovasc Surg 2011 [8, 11]
Left subclavian artery bypass first (on/off pump)	2010	Thorac Cardiovasc Surg 2012
LiquoGuard CSF drainage in CAD and TAA	2011	*No publication*
Immediate visceral reperfusion after FET anastomosis	2012	MITAT 2015 [17]
Enovia (3 Zone Graft—AAD)	2013	Charing Cross 2015 JTCVS 2020
E-vita Neo (Zone-0 FET)	2017 2020	Aortic Live 2017 CTS Net 2020

*TEVAR* thoracic endovascular aortic repair, *CSF* cerebrospinal fluid, *CAD* chronic aortic dissection, *TAA* thoraco-abdominal aortic aneurysm, *FET* frozen elephant trunk, *AAAD* acute type A aortic dissection

Over more than one decade, data was sampled and validated at Essen and got affirmation by the International E-vita Open Registry, which was initiated in 2008 by us. It was expanding from 8 to 19 participating centers over a 12-year period, with up to 1200 patient data sets demonstrating the effectiveness and durability of the device in the treatment of acute and chronic dissection as well as aneurysmal disease [10–13]

This accumulation of knowledge identified unacceptable high rates of paraplegia in chronic dissection cases [14, 15] and led to modifications of our surgical approach: reduction of the stent graft length from 15 to 13 cm, distal positioning not exceeding T-level 8 by the stent graft end, and the introduction of the LiquoGuard system (Moeller Medical GmbH, Fulda, Germany) to regulate intraspinal pressure to a maximum of 10 mmHg, by continuous automated liquor drainage throughout surgery. To prevent delayed paraplegia, the device was kept in place for 72 h postoperatively. In addition, mean arterial pressure was kept beyond 80 mmHg and the central venous pressure was reduced to below 12 mmHg. To make sure that all cerebral regions were homogeneously perfused beyond the near-infrared spectroscopy (NIRS)–controlled coverage of the anterior parts of the brain, simultaneous perfusion of all head vessels during hypothermic circulatory arrest (HCA) was started in 2009. An additional positive side effect was the fact that spinal cord nutrition by collateral blood flow from the left subclavian arterial system was augmented, and ever since then, persistent spinal cord ischemic damage became very rare (1%). This was made possible due to rerouting of the sometimes difficult-to-reach left subclavian artery (LSA), by creating an extra-anatomic left axillary artery bypass with an 8-mm graft, which was then transferred into the mediastinum via the first intercostal space and connected to a second pump [16–18] (Fig. 5A). Thus, the following strategy with extracorporeal circulation (ECC) was elaborated. In detail, routine arterial cannulation of the right axillary artery and venous cannulation of the right atrium using a dual-stage cannula were done, as well as left ventricular venting, while cooling the patient to a core temperature of 28–30 °C (Fig. 6A).

**Fig. 5:**
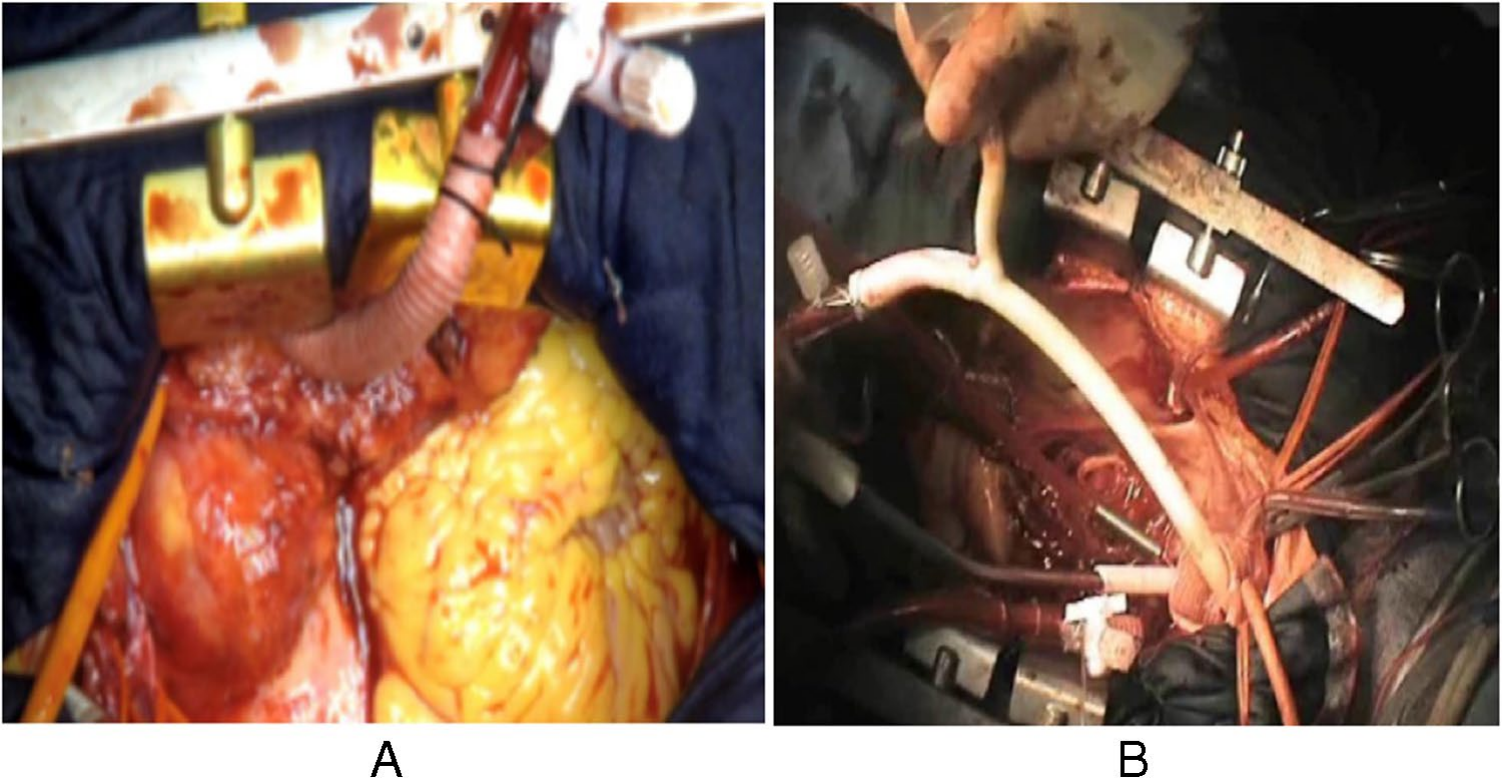
The use of a second arterial pump. **A** Extra-anatomic left axillary artery bypass with an 8-mm graft, which is transferred into the mediastinum via the first intercostal space and connected to a second pump. **B** A Foley 30 Ch balloon-tipped catheter, connected via a Y-connector to the second arterial pump, is inserted into the stented portion of the hybrid graft

**Fig. 6:**
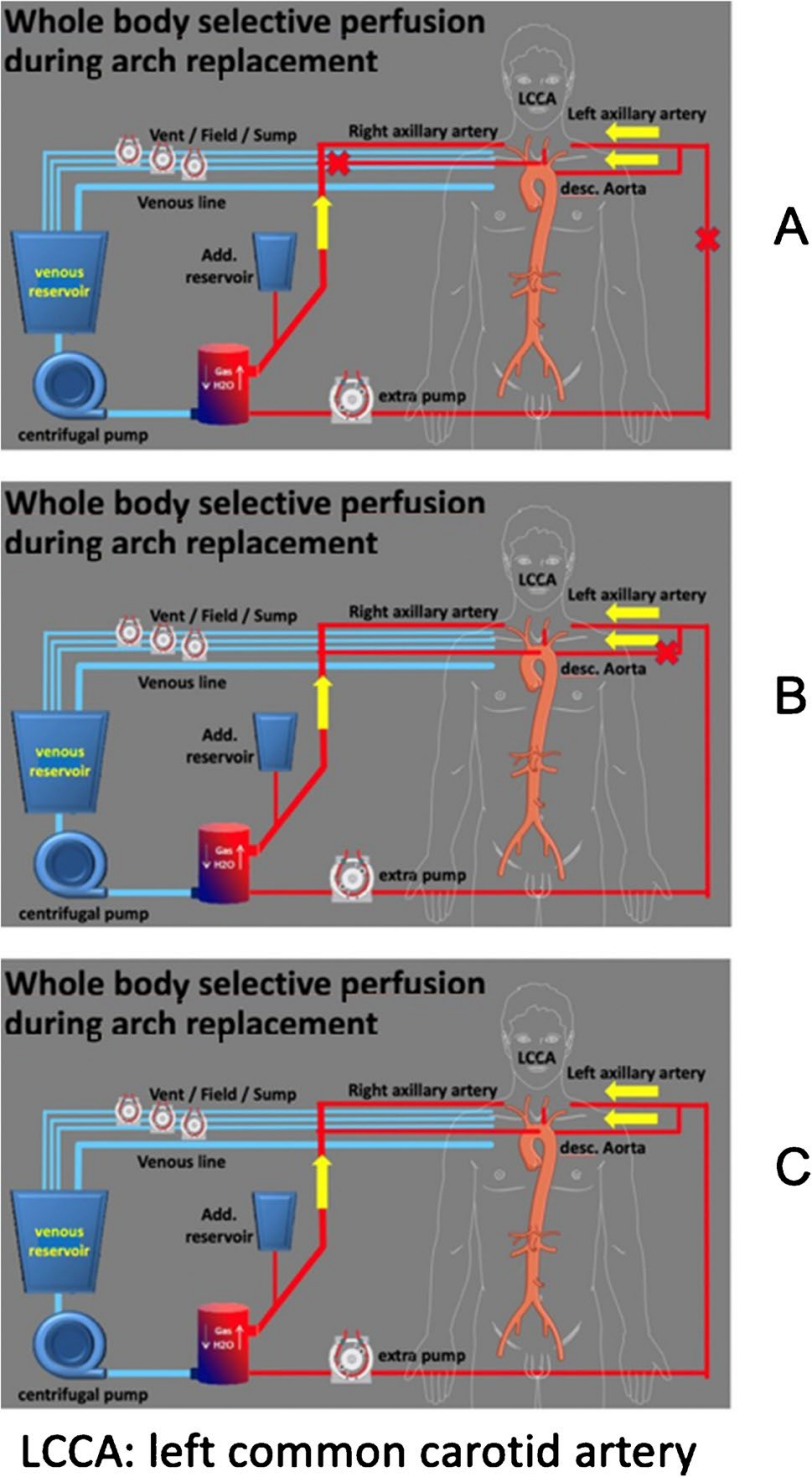
Extracorporeal circulation (ECC) during aortic arch replacement. **A** Arterial cannulation of the right axillary artery and venous cannulation of the right atrium using a dual-stage cannula. Start ECC with perfusate temperature 28–30 °C during proximal repair. **B** Start second pump, full perfusion of all 3 head vessels during hypothermic circulatory arrest (HCA) with cooling of the selective antegrade cerebral perfusion (SACP) to 22–24 °C. **C** After finishing the arch anastomosis, lower-body perfusion is started via Foley catheter into the prosthesis

The second arterial pump is connected to the left axillary artery via the extra-anatomic bypass, which is started immediately after finishing the proximal aortic repair, start of hypothermic circulatory arrest, and transection of the distal ascending aorta. Deairing and ligature of the proximal left subclavian artery precede this maneuver. Via a Y-connector, the second arm of the first arterial pump is cannulated into the left common carotid artery (LCCA); thus, full perfusion of all 3 head vessels is achieved, while cooling the perfusate to 22–24 °C (Fig. 6B). After resection of the aortic arch to zone 2, the E-vita Open Plus hybrid graft is inserted along a stiff guide wire into the TL of the descending aorta in dissection cases, or beyond the aneurysmal pathology in the distal arch guided by angioscopy. After the aortic anastomosis, a Foley 30 Ch balloon-tipped catheter, connected via a Y-connector to the second arterial pump, is inserted into the stented portion of the hybrid graft [17] (Figs. 5B and 6C). After meticulous deairing for removal of potential air bubbles obstructing the offspring of the spinal arteries, full body perfusion and slow rewarming of the patient are started. After retraction of the intussuscepted non-stented graft portion into arch position, the reconnection of the head vessels into the arch graft is done either by island- or isolated reimplantation technique. After completion of this anastomosis, the Foley catheter is removed, deairing takes place, and the arch graft is clamped for finishing the procedure by anastomosing the previously implanted ascending graft with the arch graft, while finally rewarming the patient. After cross-clamp release, the extra-anatomic bypass via the second arterial pump is transected and reimplanted to either the ascending or arch graft, where appropriate. Discontinuation of ECC with reversal of heparin by protamine application and meticulous hemostasis including thromboelastometry-guided differentiated replenishment of fibrinogen, platelets, and prothrombin complex concentrate, if required, complete surgery [19].

Though variable in our hands for either island technique or selective head vessel reimplantation into the arch portion of the E-vita Open Plus, many surgeons prefer the individual reconnection of the head vessels with a branched FET like the Thoraflex device (Vascutek, Renfrewshire, Scotland). This prompted the company to embark on the development of 3 variations of the E-vita Open Plus for additional variability and ease of implantation. With European conformity (CE) mark approval in March 2020, now a straight or triple-branched as well as a trifurcated version, all with an additional perfusion port for immediate lower-body perfusion after anastomosis either in zone 3, 2, 1, or even 0, became available (Fig. 7). With a short and surgeon-friendly application kit for fast and easy implantation, this device means a true progress in adopting the surgical approach to all kinds of pathologies and situations, minimizing the need for hypothermic circulatory arrest time. First in man implantation was performed by us during the Aortic Live Symposium in Hamburg 2017 (Table 1).

**Fig. 7:**
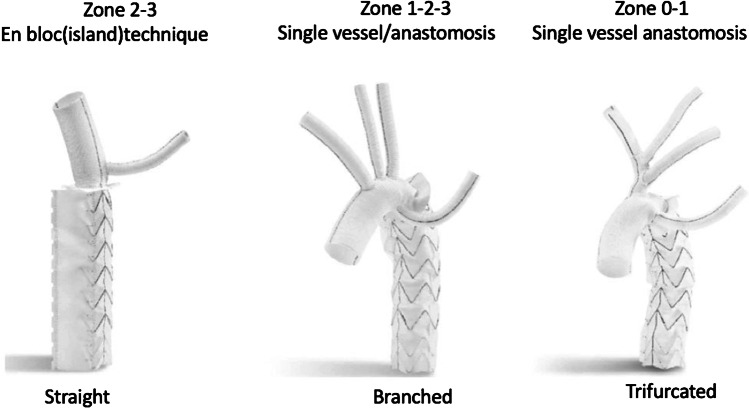
E-vita Open NEO (European conformity (CE) mark February 2020)

Stable results and positive remodeling in the stented area of more than 90% during follow-up (Fig. 8A, B) [20, 21] could be achieved. More distally, about two thirds of the patients face positive remodeling depending on the size and number of re-entry sites downstream [22]. In addition, we could prove that acute type I dissections with distal arch/proximal descending aorta re-entry sites could also be a robust indication for FET application, reducing the need for downstream re-intervention or open re-operation within 10 years in our center to 26%, in contrast to 52% after proximal repair, at a low peri-operative mortality risk [23].

**Fig. 8:**
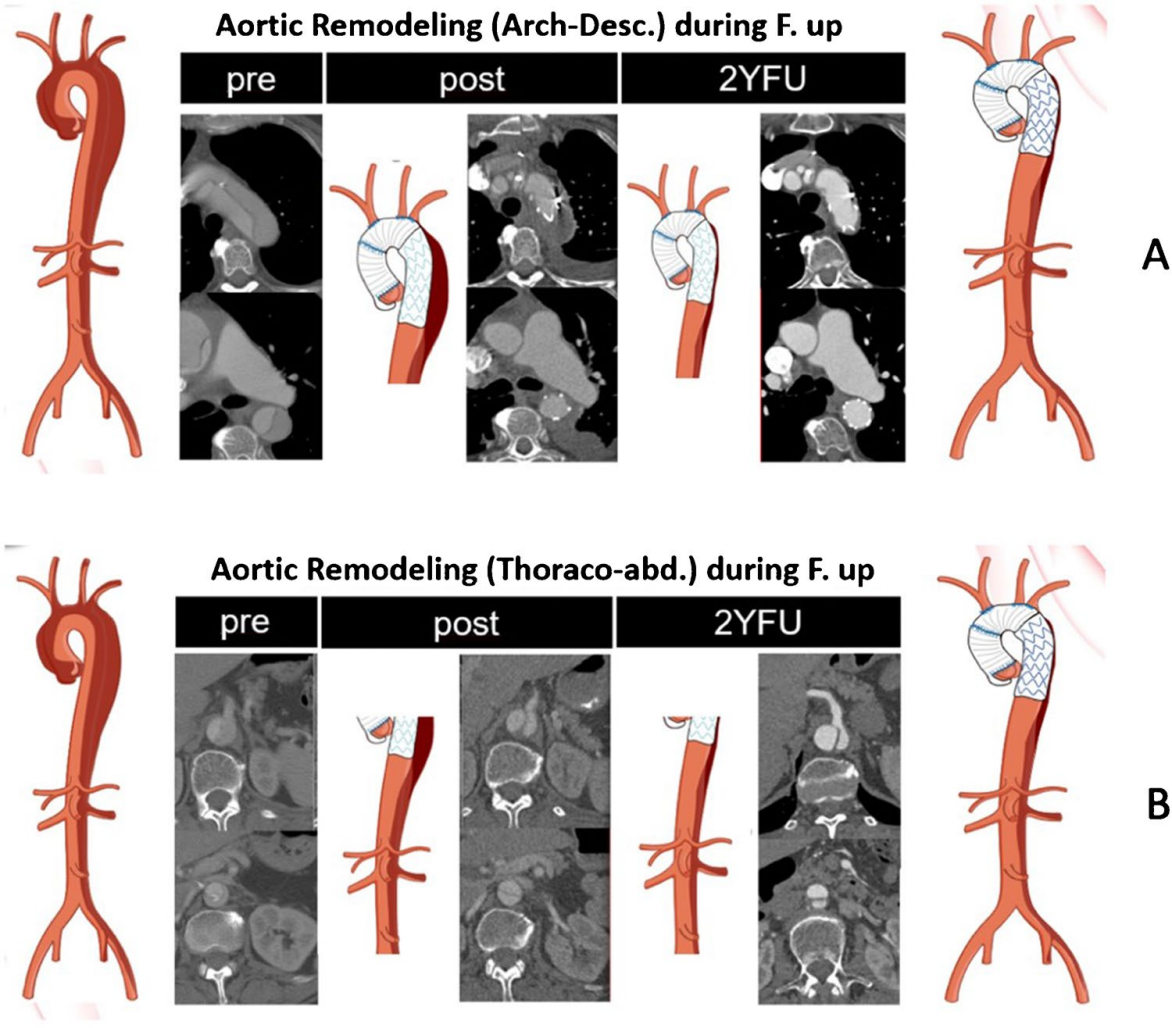
Aortic remodeling during follow-up. **A** Aortic arch and descending aorta. **B** Thoraco-abdominal aorta

It is a well-known fact that the implantation of a stent graft into the descending aorta carries the inherent risk of provoking a distal stent–induced new entry (DSINE). Learning from the past with too much oversizing of the stent graft in aortic dissection cases by taking the total aortic diameter as a guideline, in some cases, subsequent rupture, penetration, and fistula formation were experienced. This prompted our more conservative approach with taking the length of the separating lamella between the TL and false lumen (FL) as a measure for the graft size to be chosen, regardless of whether dissection was acute or chronic. In aneurysmal cases, a maximum of 10% oversizing was accepted taking care to fully cover the pathology with a 2–3-cm overlap into the non-aneurysmal distal part of the descending aorta. This resulted in a DSINE incidence of 2.9% in acute type A aortic dissection (ATAAD) cases [24]. In chronic aortic dissection (CAD) cases treated either by FET or thoracic endovascular aortic repair (TEVAR), the reported DSINE incidence ranges from 6.5 to 28% within 3 years [24–27].

To probably further reduce the incidence of DSINE, the z-stent orientation within the E-vita Open NEO device was switched from tip-to-tip spring orientation to a tip-to-valley constellation, thus creating better alignment to curved structures, which probably will reduce the radial recoil force of the stented portion. In addition, the distal 2 springs were positioned inside the fabric. Together with the non-oversizing concept, both components are key to the reduction of local wall stress to the separating membrane in dissection cases (Fig. 9).

**Fig. 9:**
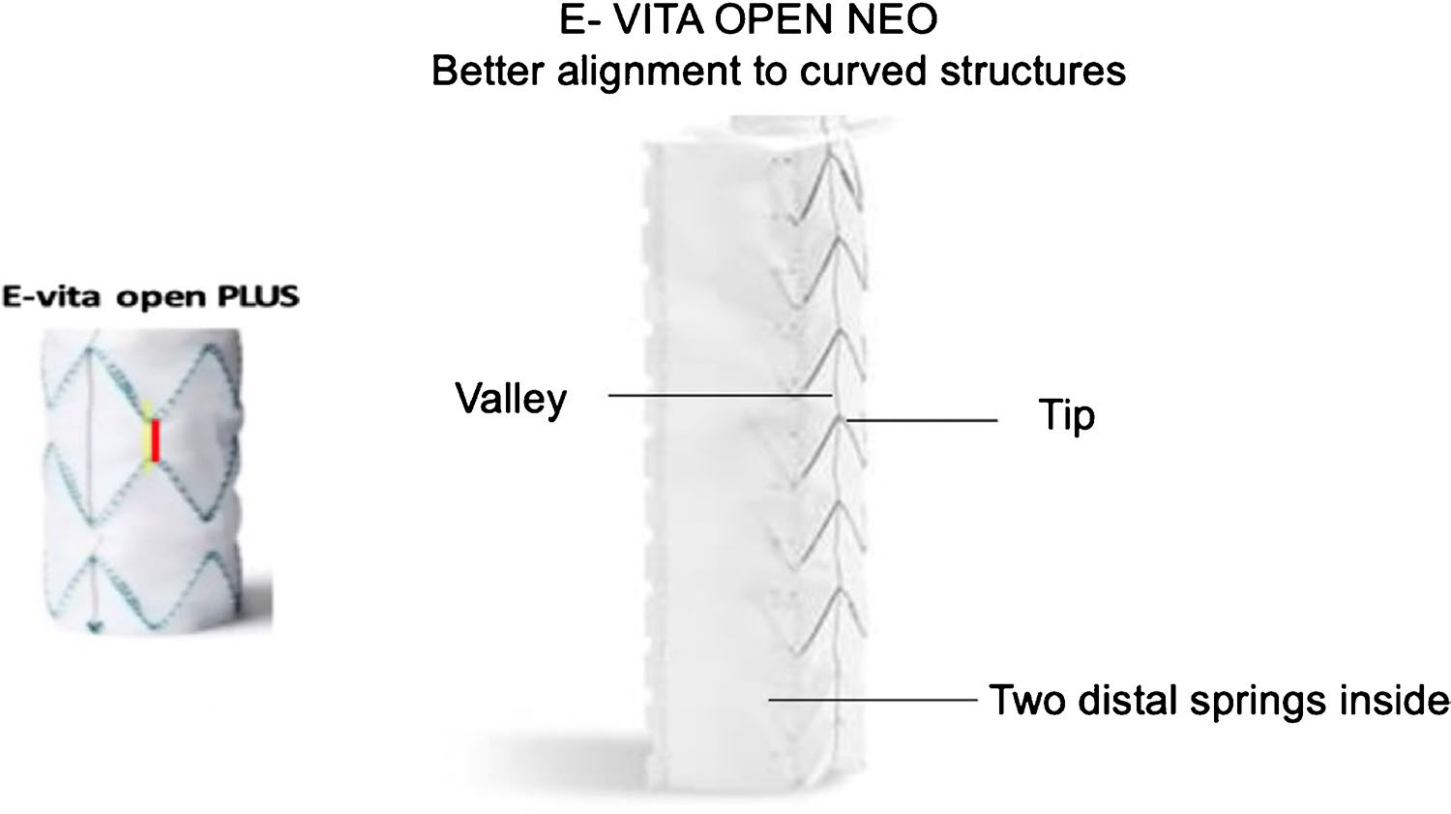
E-vita Open NEO design in comparison to E-vita Open Plus

More recently, oozing through the pores of the E-vita Open NEO was made an issue, although the weaving process of the new graft was identical to the previous one, which had not caused this phenomenon for over a decade [28]. However, modern coagulation management using rotational thromboelastometry provides important information about hemostasis, particularly in regard to fibrin polymerization and platelet function, and is available in most tertiary-care institutions. Accordingly, cryoprecipitate can be prepared timely at cross-clamp release, if fibrinogen concentrate is not available. Adequate fibrin and platelet contributions to the clot firmness are essential for the formation of an internal fibrin layer to seal the prosthesis. Thus, volume overload using fresh frozen plasma infusions, with its very low fibrinogen contents, can be specifically avoided [19, 29].

Other groups also report comparable results with different FET devices like the branched Thoraflex graft and the Japanese version (Japan LifeLine, Tokyo, Japan) [30, 31]. The Chinese Cronus device (MicroPort Medical, Shanghai, China), though available since 2004, is mainly implanted in China with reported excellent results [32]. Cronus, as well as Frozenix, requires the addition of a second arch graft to be anastomosed to the cut FET, either in zone 2 or 3.

Overall, the 15-year experience with the E-vita Open and Open Plus graft, with all the demonstrated influential strategic innovations, can serve as a reference for comparison with other approaches with different FET devices. The recently reported results, especially with the last 177 patient cohort, are quite satisfying with their 11% 30-day mortality, 1% persisting spinal cord injury, and 7% new stroke occurrence [33].

With this prerequisite in mind, the E-vita Open NEO represents the next generation of perfected hybrid prostheses for the treatment of complex thoracic aortic diseases encompassing the arch and adjacent structures. Thus, a new era starts and the upcoming less invasive endovascular treatment options, for this kind of pathologies, have to be measured with this gold standard and its proven long-term stability.
